# On the Optimization of a Centrifugal Maglev Blood Pump Through Design Variations

**DOI:** 10.3389/fphys.2021.699891

**Published:** 2021-06-18

**Authors:** Peng Wu, Jiadong Huo, Weifeng Dai, Wei-Tao Wu, Chengke Yin, Shu Li

**Affiliations:** ^1^Artificial Organ Technology Laboratory, School of Mechanical and Electric Engineering, Soochow University, Suzhou, China; ^2^School of Mechanical Engineering, Nanjing University of Science and Technology, Nanjing, China; ^3^Institute for Medical Device Control, National Institutes for Food and Drug Control, Beijing, China

**Keywords:** centrifugal blood pump, optimization, computational fluid dynamics, turbulence, hemolysis

## Abstract

Centrifugal blood pumps are usually designed with secondary flow paths to avoid flow dead zones and reduce the risk of thrombosis. Due to the secondary flow path, the intensity of secondary flows and turbulence in centrifugal blood pumps is generally very high. Conventional design theory is no longer applicable to centrifugal blood pumps with a secondary flow path. Empirical relationships between design variables and performance metrics generally do not exist for this type of blood pump. To date, little scientific study has been published concerning optimization and experimental validation of centrifugal blood pumps with secondary flow paths. Moreover, current hemolysis models are inadequate in an accurate prediction of hemolysis in turbulence. The purpose of this study is to optimize the hydraulic and hemolytic performance of an inhouse centrifugal maglev blood pump with a secondary flow path through variation of major design variables, with a focus on bringing down intensity of turbulence and secondary flows. Starting from a baseline design, through changing design variables such as blade angles, blade thickness, and position of splitter blades. Turbulent intensities have been greatly reduced, the hydraulic and hemolytic performance of the pump model was considerably improved. Computational fluid dynamics (CFD) combined with hemolysis models were mainly used for the evaluation of pump performance. A hydraulic test was conducted to validate the CFD regarding the hydraulic performance. Collectively, these results shed light on the impact of major design variables on the performance of modern centrifugal blood pumps with a secondary flow path.

## Introduction

In recent years, ventricular assist devices (VAD, also called blood pumps) have gradually replaced heart transplantation as an effective treatment for heart failure (Bellumkonda and Bonde, 2012; Molina and Boyce, 2013; Magruder et al., 2016; Cui et al., 2018; Gaffey et al., 2018; Kawabori et al., 2018; Shahreyar et al., 2018). However, after the implantation of these VADs, adverse effects related to blood mechanical damage, such as bleeding and stroke and thromboembolic events, were often reported (Birschmann et al., 2014; Maltais et al., 2016; Netuka et al., 2016; Mehra et al., 2017; Shah et al., 2017). Blood damage and its complications have become the pain point in the clinical application of VADs and mechanical circulatory support devices, as well as other blood contacting medical devices (Bluestein et al., 2002; Ge et al., 2008; Polaschegg, 2009; Faghih and Sharp, 2019). The main cause of blood mechanical damage is the complex geometric structure and mechanical movement in blood pumps, which makes blood cells experience non-physiological stress which is much higher than normal physiological stress (Li et al., 2013). Turbulence and secondary flow will further increase the blood damage (Wu et al., 2020). Moreover, mechanical bearings result in friction and heating, bringing secondary damage to blood; the flow dead zone around mechanical bearing increases thrombosis risk. Maglev bearings avoid mechanical contact, friction and dynamic sealing; thus, it can avoid blood damage and flow dead zone. The clearance of Maglev bearing is can be wide, resulting in low stress, reduced blood damage and good biocompatibility. Therefore, maglev blood pump is the trend of blood pumps (Fraser et al., 2011). Nonetheless, good hydraulic and blood compatibility designs are still very important for maglev pumps. Blood damage of maglev pumps may still be higher than that of blood pumps using mechanical bearings. Sobieski et al. (2012) conducted hemolysis tests of the maglev blood pump Centrimag and Maquet Rotaflow which uses mechanical bearings, found that the hemolysis level of Centrimag is higher than Rotaflow for two test conditions. Centrimag was designed before 2005, there have been many studies on its flow field and blood compatibility in recent years. Centrimag features relatively large clearance (2∼3 mm) to reduce the stress. However, the secondary flow intensity would increase considerably, and hemolytic performance is not as low as one might be expected for a maglev blood pump (Sobieski et al., 2012). Centrifugal blood pumps normally feature secondary flow path beneath the impeller (Gregory et al., 2017; Zhang et al., 2020), to avoid flow stagnation zones and the resulting risk of thrombosis. For extracorporeal blood pumps with high-pressure heads, the flow rate in the secondary flow path can reach up to approximately 70% of the main flow rates. The secondary flow intersects the main flow near the leading edge, bringing significant disturbance to the main flow, making the flow more disorderly and turbulent. Turbulence is one of the primary causes of hemolysis (Kameneva et al., 2004; Hund et al., 2010; Wu et al., 2019). Although there have been some studies that proposed hemolysis models to account for turbulence effects (Wu et al., 2019, 2020), the credibility of hemolysis prediction in turbulence remains an open question.

In recent years, many efforts have been devoted to the optimization of blood pumps, mainly focusing on improving hydraulic efficiency and blood compatibility in blood pumps with mechanical bearings. Wu et al. (2010) numerically investigated the influence of tip clearance on the hemolytic performance of a centrifugal blood pump, found a tip clearance of 100 microns is the most optimal. Song et al. (2010) studied the effects of straight blades, forward-curved blades, and backward-curved blades on the hemolytic performance through numerical simulations showed that the backward-curved blades have better hemolysis performance. Caglar et al. (2018) studied the influence of the blade wrap angle on the hemolytic and hydraulic performance of a centrifugal blood pump, identified a best wrap angle of 120°. Wiegmann et al. (2017) investigated the effects of tip clearance, the number of blades, and shroud design on hemodynamics and hydraulic performance of a centrifugal pump. Nonetheless, little scientific study has been published concerning optimization and experimental validation of centrifugal blood pumps with secondary flow paths, which feature high intensity of turbulence and secondary flows.

The purpose of this study is to optimize the hydraulic and hemolytic performance of a centrifugal maglev blood pump with a secondary flow path through variation of major design variables. The effect of alterations in volute design, blade installation angles, thickness and diameter of the splitter blades was investigated. Computational fluid dynamics (CFD) combined with hemolysis models were mainly used for the evaluation of pump performance. Hydraulic tests were conducted to validate hydraulic performance predicted by CFD. In view of high turbulence intensity and the inadequacies of current hemolysis models in the prediction of hemolysis in turbulence, indicators of turbulence intensity such as turbulent eddy viscosity and Q-criteria were also considered. Collectively, these results shed light on the impact of major design variables on the performance of modern centrifugal blood pumps.

## Materials and Methods

### Baseline Pump Design

**FIGURE 1:**
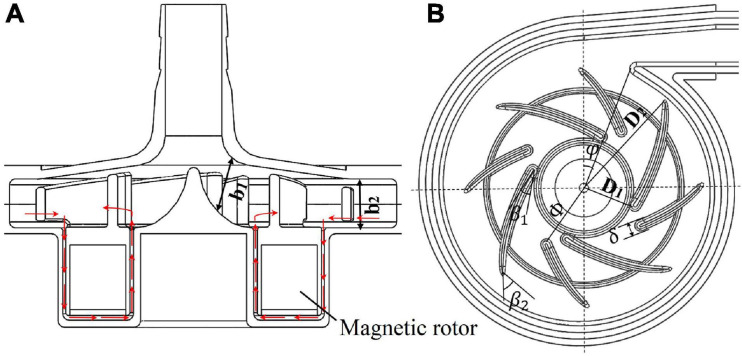
**(A)** Schematic of the maglev blood pump model, red arrows indicate flow direction of the secondary flow path; **(B)** baseline design of the impeller and volute, showing main design parameters.

The baseline design was based on a maglev blood pump previously developed in our group. The schematic of the pump head is shown in Figure 1A. The cavity which accommodates the magnetic rotor was combined with the rotor into an integral structure. The shell of the pump head and the cavity form a secondary flow path. The total blade number was determined as eight, with four splitter blades. The baseline pump head was designed using the speed coefficient method combined with the centrifugal pump one-dimensional design theory. The design point was set as: rpm at 3,500 r/min, pressure head of 360 mmHg, and flow rate at 5 L/min. The main design parameters of the impeller (as shown in Figure 1) were determined accordingly and are shown in Table 1.

**TABLE 1:** Design parameters of the pump impeller.

**Parameter**	**Symbol**	**Value**
Inlet diameter of impeller	*D*_*1*_	9.6 mm
Outlet diameter of impeller	*D*_*2*_	54 mm
Inlet width of blade	*b*_*1*_	7.3 mm
Outlet width of blade	*b*_*2*_	7 mm
Blade inlet angle	β_*1*_	14°
Blade outlet angle	β_*2*_	41.7°
Number of blades	Z	8

### Design Variations

The design variables considered in the study of design variations include blade inlet and outlet angles (β_1_,β_2_), blade thickness (δ) and radius of the leading edge of splitter blades (Φ), as shown in Figure 1B. The inlet and outlet blade angles are not independent variables. They are related to each other by the wrap angle, and also by constraints such as keeping the blade streamlined. Considering the influence of the secondary flow path, the inlet blade angle was increased to 63.3°, and the outlet blade angle was determined as 62.9°, to keep the blade streamlined one hand, and better match the volute design on the other hand. Two levels were also made for δ and Φ. This led to four pump models, as shown in Table 2.

**TABLE 2:** Design variables and variations.

**Model**	**(β_1_,β_2_)**	**δ**	**Φ**
1	14°, 41.7°	1.5 mm	15 mm
2	63.3°, 62.9°	1.5 mm	15 mm
3	63.3°, 62.9°	2 mm	15 mm
4	63.3°, 62.9°	2 mm	13.5 mm

### CFD Analysis

Computational fluid dynamics simulations were employed to evaluate the hydraulic and hemolytic performance of all the models of blood pumps. A cylindrical surface was placed downstream of the blade trailing edge, which acts as an interface between the rotating regions of the impeller and secondary flow path, and the rest stationary regions. A hybrid mesh was employed. The grid was tetrahedral with 5 prism layers, generated using Ansys meshing (Ansys, Inc., Canonsburg, PA, United States). A grid sensitivity analysis was also performed for model 1, with three grids of 5.2 million, 10.4 million and 26.5 million, respectively (as shown in Table 3). The rotational speed was set as 3,500 RPM, and inlet flow rate was set as 5L/min for grid sensitivity analysis.

**TABLE 3:** Details of mesh for grid sensitivity analysis.

**Mesh**	**Cells (×10^6^)**	**Mean y^+^ on impeller surface**	**Maximumy^+^**
Coarse	5.0	1.11	3.53
Middle	10.4	0.82	2.75
Fine	26.5	0.31	1.17

The CFD simulations were performed using the commercial software Ansys Fluent. The flow rate of 5 L/min was applied at the inlet. The blood was regarded as a Newtonian fluid, and the viscosity was taken as 3.5 mPa⋅s. For each pump model, the rotational speed of the rotor was adjusted through iterative computations to meet the targeted pump head of 360 mmHg. A SIMPLE method was employed to solve the incompressible N-S equations. Turbulence was modeled using the RNG k-ε model. The steady “frame motion” approach was used to couple the rotating and stationary regions. Convergence criteria was set that the residuals of all equations drop below 10^–6^.

### Hemolysis Predictions

Four hemolysis models were employed in this study to estimate hemolysis level of the blood pumps, three of which were stress-based models, which relate hemolysis to effective stress τ and exposure time t through a power-law relationship (Heuser and Opitz, 1980; Giersiepen et al., 1990; Song et al., 2003; Zhang et al., 2011).

(1) $$HI\left(\%\right)=\frac{h\,b}{H\,b}\times 100=C\tau_{\text{eff}}^{\alpha }t^{\beta }$$

where HI(%) is the hemolysis index in percentage, τ_eff_ is the effective stress and a scalar quantity, Hb is the total hemoglobin concentration, *hb* represents the increase in plasma free hemoglobin; *C*, α, and β are empirical constants. Three widely used power law models were employed in this study. The effective stress τ_eff_ is calculated by adding up viscous and Reynolds stress. Table 4 lists the empirical constants of the three stress-based power-law models employed in this study.

**TABLE 4:** Empirical constants of the stress-based power-law hemolysis models.

**Model**	***C***	**α**	**β**
GW	3.620 × 10^−5^	2.4160	0.7850
HO	1.800 × 10^−4^	1.9910	0.7650
TZ	1.228 × 10^−5^	1.9918	0.6606

*GW, model of Giersiepen et al. (1990). The constants of the HO model were originally derived from Heuser and Opitz (1980) by Song et al. (2003). TZ, model of Zhang et al. (2011).*

One can note that the exposure time in Eq. 1 is nonlinear. It would be incorrect to consider that the hemolysis at the domain outlet is the sum of the local hemolysis, because of the nonlinear dependency in time. This problem was avoided by introducing *hb*′ as a scalar variable equal to *hb*^1/β^. Then Eq. 1 can be reorganized into a Eulerian scalar transport equation (Garon and Farinas, 2011).

(2) $$\frac{d\,\left(h\,b^{\prime }\right)}{d\,t}+v\,\rho \cdot \nabla \left(h\,b^{\prime }\right)=C,$$

where C  is the source term defined as,

(3) $$C=\rho \,{\left(H\,b\cdot c\cdot \tau_{\text{eff}}^{\alpha }\right)}^{1/\beta }$$

In addition to the stress-based model, an energy-dissipation-based (EDB) power-law model proposed by Wu et al. (2010, 2020) was also employed. Compared with stress-based models, this model can improve the prediction of hemolysis for a wide range of flow conditions, especially turbulent flows. The EDB model reads:

$$HI\left(\%\right)=\left[a\cdot {\left(\varepsilon \cdot 10^{-7}\right)}^{2}+b\cdot \varepsilon \cdot 10^{-7}\right]/$$

(4) $$[{\left(\varepsilon \cdot 10^{-7}\right)}^{2}+c\cdot \;\varepsilon \cdot 10^{-7}+d]\cdot t$$

where the constants were determined as: *a* = −1,*b* =  15.88,*c* = −36.44,*d* =  326.7, ε; is energy dissipation rate and the sum of viscous dissipation rate ε_vis_ and turbulent dissipation rate ε_turb_. Please refer to Wu et al. (2019, 2020) for the definition and calculation of these two variables.

The HI(%) was then calculated from the mass-weighted average of *hb* at the outlet of the device divided by *Hb*. Hemolysis predictions started after flow simulations had converged, with all flow variables frozen.

### Validation

**FIGURE 2:**
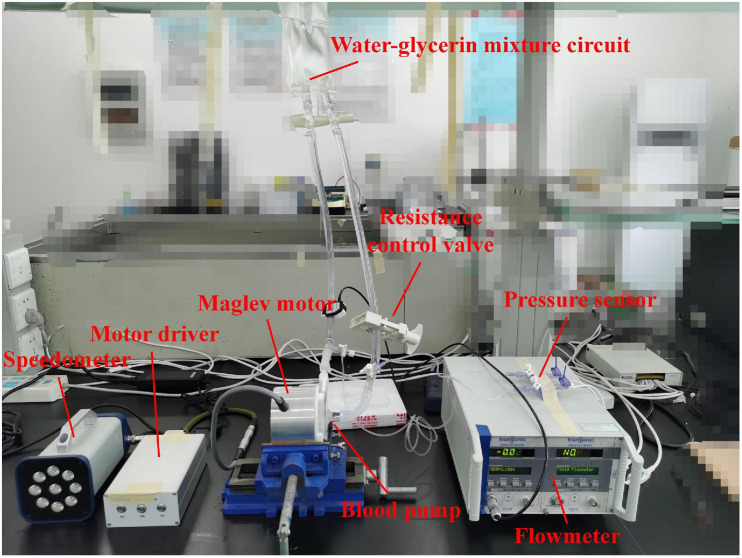
Schematic of hydraulic experiment.

Hydraulic tests were carried out to verify the accuracy of CFD simulations and obtain the hydraulic performance of the blood pump. The hydraulic test rig was shown in Figure 2. The pump head was made from photosensitive resin by 3D printing. A water-glycerol mixture was used to simulate the blood viscosity of 3.5 mPa⋅s at room temperature of 24°C. The flow rate was measured with an in-line ultrasonic flow probe (Transonic, Inc., Ithaca, NY, United States). Pressures were acquired via disposable pressure transducers at a small distance to the inflow and outflow cannula. A resistance regulating valve was employed to control the flow rates. Four rotational speeds were chosen ranging from 1,700 rpm to 2,800 rpm. Twenty operating points were tested, with pressure head and flow rate recorded.

## Results

### Grid Sensitivity Analysis

The results of grid sensitivity analysis are shown in Table 5. Pressure head and hemolysis index were compared to the results predicted with the fine mesh. The HO model was used for hemolysis prediction. For the “Middle mesh,” the error of pressure head was within 5%, and the error of hemolysis was within 1%. Therefore, a grid of around 10 million is the most appropriate, with results sufficiently resolved and relatively low computational cost compared with the finer mesh. Therefore, the number of grid elements for different pump models was kept at around 10 million.

**TABLE 5:** Results of grid sensitivity analysis.

**Mesh**	**Cells (×10^6^)**	**P(mmHg)**	**Error of P (%)**	**Error of HI (%)**
Coarse	5.0	420	5.1	1.28
Middle	10.4	424.5	4.1	0.3
Fine	26.5	442.7	/	/

*P, predicted pressure head; error of P (%), defined as —P−*P*_0_|/*P*_0_, where *P*_*0*_ is the pressure head predicted with the fine mesh; HI(%), hemolysis predicted using the HO model; error of HI(%), defined as —HI−*HI*_0_|/*HI*_0_  , where *HI*_*0*_ is the HI predicted with the fine mesh.*

### Blade Inlet and Outlet Angles

**FIGURE 3:**
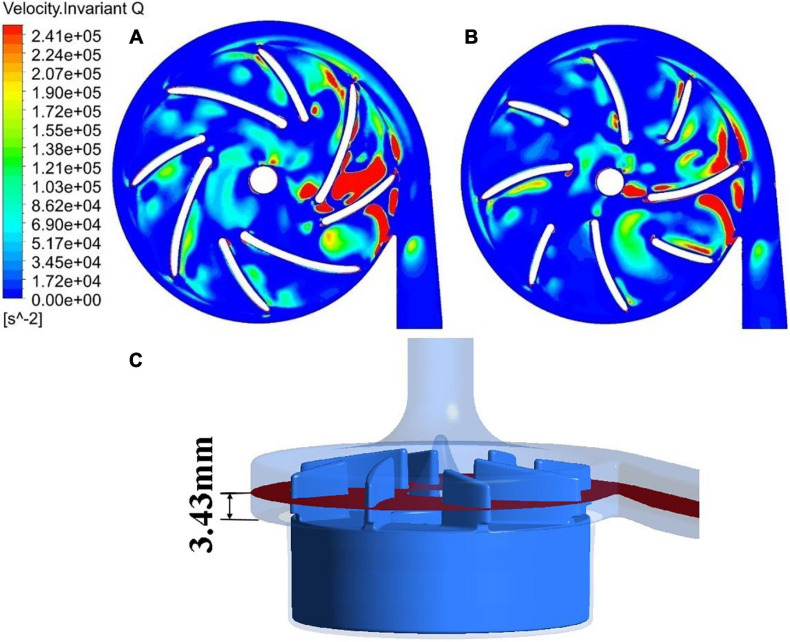
Contours of invariant Q of velocity: **(A)** model 1 [blade angles of (14°, 41.7°)]; **(B)** model 2 [blade angles of (63.3°, 62.7°)]. **(C)** The plane where the 2D contours in this study were taken from.

**FIGURE 4:**
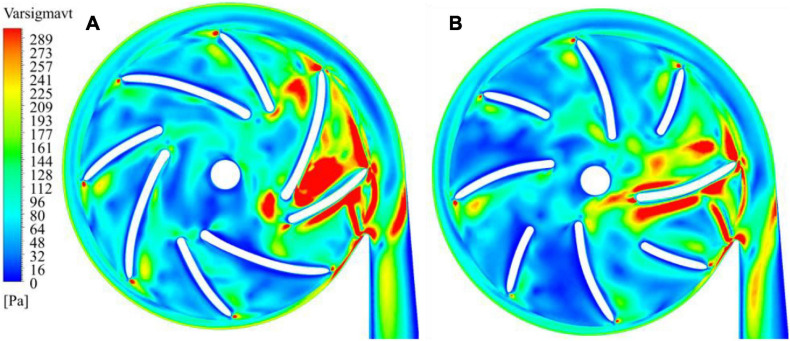
Contours of effective stress: **(A)** model 1; **(B)** model 2.

The blade inlet and outlet angles (β_1_,β_2_) were changed from (14°, 41.7°) of model 1 (baseline design) to (63.3°, 62.7°) of model 2. This subsection shows the results of model 1, in comparison with model 2. Invariant Q of velocity (Q-criteria) has been widely used as an indicator of vortex intensity. According to the Q criteria, positive *Q* values means that there is vortex generation. Vortex intensity becomes stronger as *Q* value increases. As shown in Figure 3, vortex intensity increases dramatically in the blade passages near the tongue for both pump models. Furthermore, the vortex intensity of model 2 is significantly lower than model 1. Figure 4 shows the contours of effective stress for both pump models. The regions of high effective stress are consistent with the regions of high *Q* value. Both Q and effective stress are important indicators of turbulence intensity. The results show that turbulence intensities in the blade passages near the tongue decreased considerably for model 2 compared with model 1.

**FIGURE 5:**
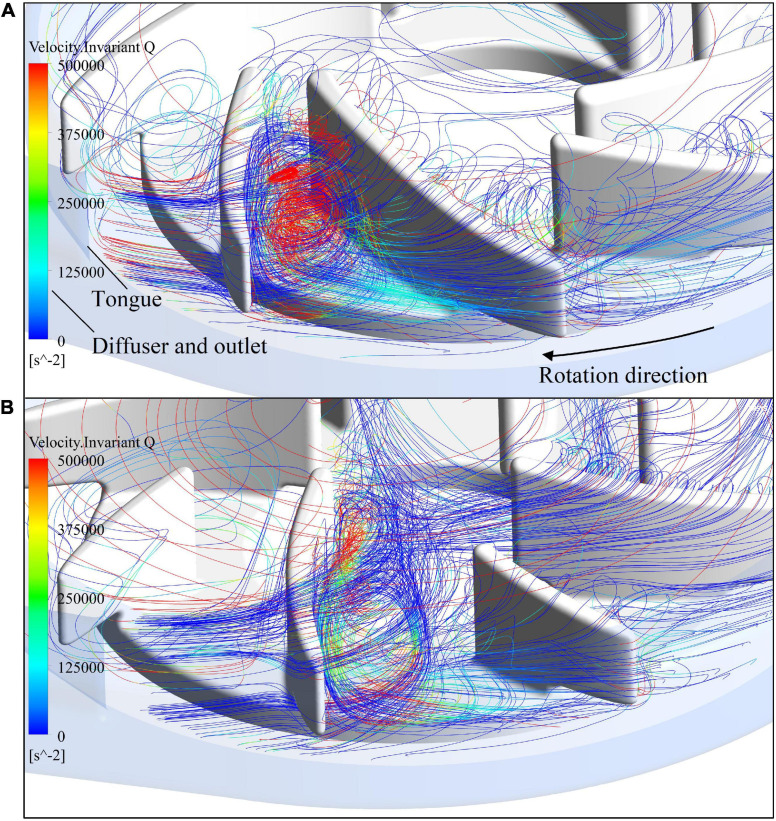
Streamlines in the blade passage near the tongue, colored by invariant Q of velocity: **(A)** model 1; **(B)** model 2.

Figure 5 shows streamlines in the blade passage near the tongue, colored by invariant Q of velocity. As the blade approached the tongue, the leakage flow was enhanced by the impingement of the exit flow from the blade passage on the tongue. It passed the tip of the blade and formed a large secondary vortex in the adjacent blade passage. Figure 5 shows the intensity of this vortex has dropped substantially for model 2 compared with model 1, in line with what has been observed in the 2D contours.

The hydraulic efficiency was also improved, from 19.5% of model 1 to 21.0% of model 2. A new variable, *HI*_diff_, was introduced to represent the overall change of hemolysis, defined as:

(5) $$H\,I_{\text{diff}}=\left(\frac{H\,I_{\text{GW}}^{\prime }}{H\,I_{\text{GW}}}+\frac{H\,I_{\text{HO}}^{\prime }}{H\,I_{\text{HO}}}+\frac{H\,I_{\text{TZ}}^{\prime }}{H\,I_{\text{TZ}}}+\frac{H\,I_{\text{WU}}^{\prime }}{H\,I_{\text{WU}}}\right)/4-1,$$

where the primes represent the hemolysis indices of the new pump model. Here, the *HI*_diff_ is −15.83%, a significant reduction of hemolysis level compared with model 1. It can be concluded that with the new set of blade angles, both the hemolytic and hydraulic performance of the blood pump were greatly improved.

### Blade Thickness

**FIGURE 6:**
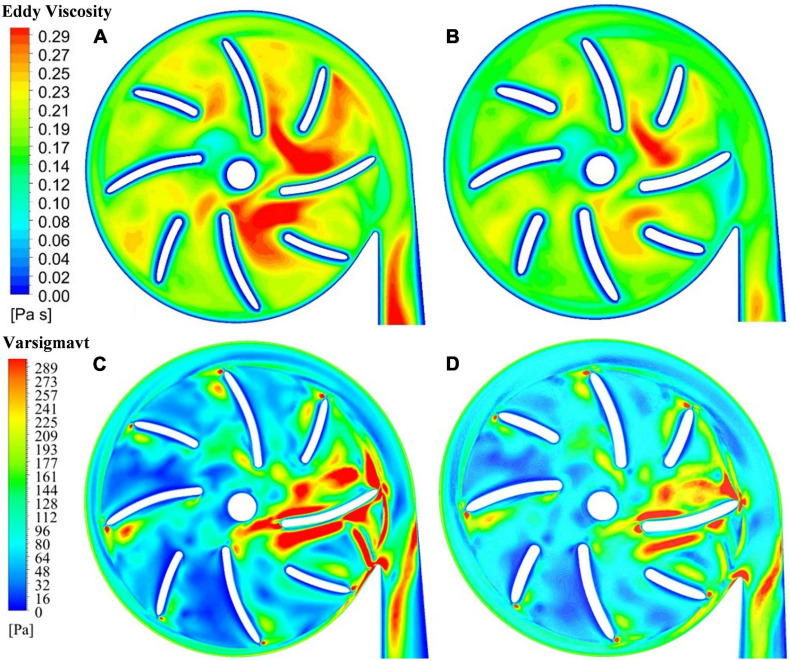
Contour of turbulent viscosity: **(A)** model 2 (blade thickness of 1.5 mm); **(B)** model 3 (blade thickness of 2 mm); contour of effective stress: **(C)** model 2 (blade thickness of 1.5 mm); **(D)** model 3 (blade thickness of 2 mm).

The blade thickness δ was changed from 1.5 mm of model 2 to 2 mm of model 3. This subsection compares the results of model 2 with model 3. Turbulence viscosity is an important indicator of turbulence intensity. Figures 6A,B show the contours of turbulent viscosity for the two pump models. It can be clearly seen that the turbulent viscosity inside pump model 3 is considerably higher than blood pump is larger than pump model 2. Thus, the increase of blade thickness brought down turbulent intensities inside the pump model, at both the blade passages and outlet.

This is also supported by the contours of effective stress. Figures 6C,D show the effective stress is significantly reduced with the increase of blade thickness from 1.5 mm to 2.0 mm. The hydraulic efficiency was increased from 21.0% of model 2 to 21.7% of model 3, while *HI*_diff_ is −10.98%, a significant reduction of hemolysis compared with model 2. Thus, with the increase of blade thickness from 1.5 mm to 2.0 mm, both the hemolytic and hydraulic performance of the blood pump were improved.

### Position of Splitter Blade

**FIGURE 7:**
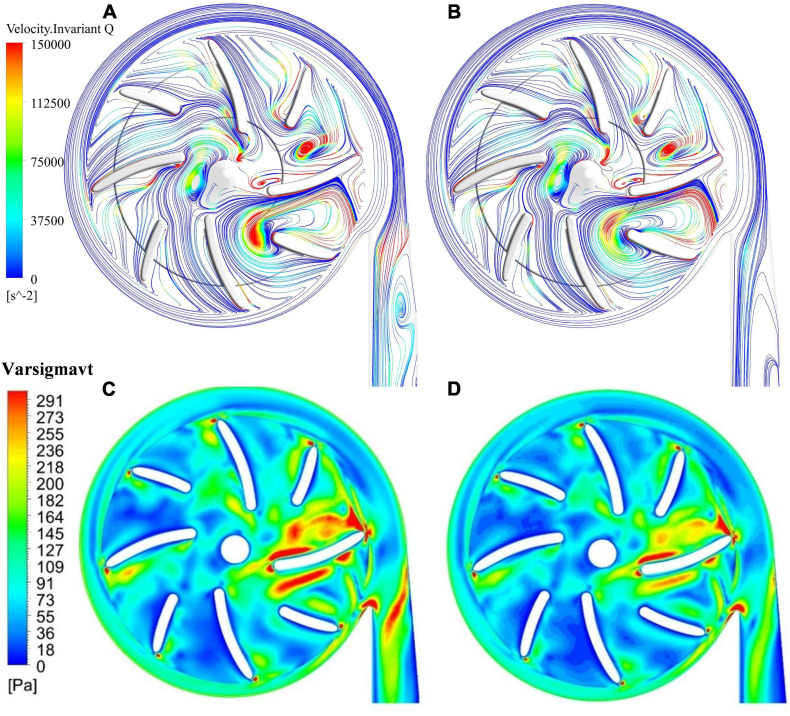
Streamlines, colored by invariant Q of velocity: **(A)** model 3 (radius of the leading edge of splitter blades Φ = 15 mm); **(B)** model 4 (Φ = 13.5 mm); contours of effective stress: **(C)** model 3; **(D)** model 4.

Splitter blades were often used to regulate the flow field and improve hydraulic performance in traditional turbomachines. In this study, two positions of splitter blades and their influence on the hydraulic and hemolytic performance of blood pumps were investigated, with a radius of the leading edge of splitter blades being 15 mm (model 3) and 13.5 mm (model 4), respectively. The flow field distribution at the outlet of the impeller and the inlet of the volute. Figures 7A,B show the surface streamlines of the two model pumps, colored by invariant Q of velocity. It can be observed that the vortex strength prior to the lead edge of the splitter blades near the tongue has been reduced. The flow separation at the diffuser was also weakened. Figures 7C,D show the effective stress contours of the two pumps models. The effective stress was reduced in the blade passages near the tongue for model 4. Therefore, it is beneficial for the suppression of the secondary flows to extend the splitter blades further upstream.

The hydraulic efficiency was increased from 21.7% of model 3 to 22% of model 4, while *HI*_diff_ is −5.63%, a notable reduction of hemolysis level compared with model 3. Thus, both the hemolytic and hydraulic performance of the blood pump were improved with an extension of the splitter blade toward upstream.

### Hydraulic Experiment

**FIGURE 8:**
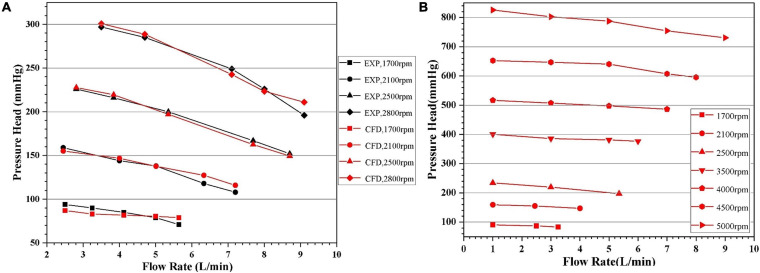
Head-flowrate (H-Q) curves predicted by CFD: **(A)** in comparison with experiment for certain rotational speeds, with the black and red symbols representing experimental and CFD data, respectively; **(B)** with pressure head up to 800 mmhg.

Pressure head (H) and flowrate (Q) of pump model 4 at four different rotational speeds (1,700 rpm, 2,100 rpm, 2,500 rpm, and 2,800 rpm) were experimentally tested. Five flow rates were selected for each speed. CFD computations of these experimental conditions were carried out. Additional CFD computations were also conducted with rotational speeds up to 5,000 rpm and pressure head above 800 mmhg to get a more complete picture of the pump performance. These H-Q curves were shown in Figure 8. It can be observed that as Q increased, the H gradually decreased. The H-Q curves are typical of centrifugal pumps, and quite flat. The requirements of hydraulic design of centrifugal blood pumps were well met. The CFD results agree well with experimental results, with standard deviations within 5% and maximum error within 10%. Thus, it is justified to rely on CFD during the optimization process.

### Summary of Design Optimization

**FIGURE 9:**
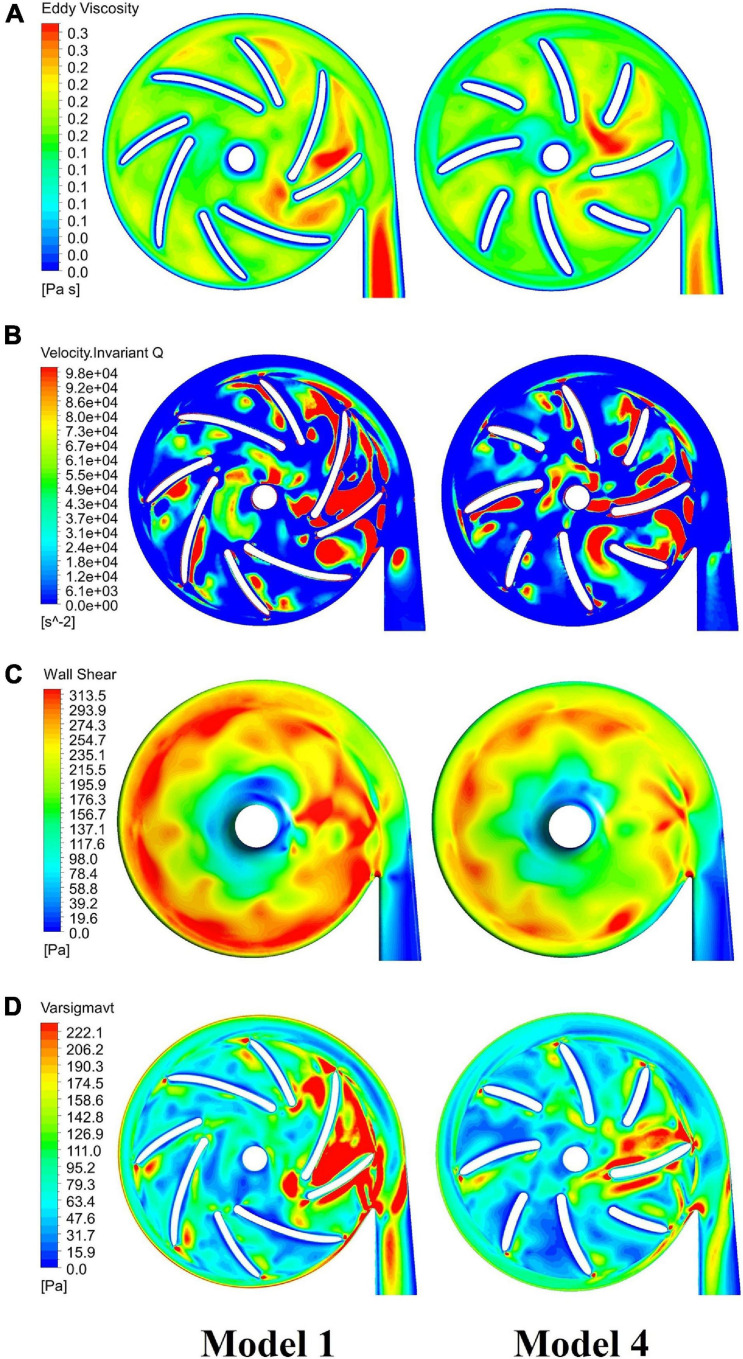
Comparison of major indicators of flow field in blood pumps: baseline pump (model 1, left column) and optimized pump (model 4, right column), including: **(A)** eddy viscosity; **(B)** invariant Q of velocity; **(C)** wall shear stress at the casing; **(D)** effective stress.

Figure 9 compares contours of major indicators of flow field in pump models before and after optimization, including eddy viscosity, invariant Q of velocity, wall shear stress at the casing and effective stress. Table 6 summarized the design variations, with variations of major metrics of pump performance, i.e. hemolysis and efficiency. The overall level of these quantities has been reduced considerably, leading to a significant improvement of both hydraulic and hemolytic performance of the pump model.

**TABLE 6:** Summary of design variations.

**Design variable**	**The pump models involved**	***HI*_diff_**	**Efficiency η**
Blade angles	Model 1 (baseline design)	−15.83%	19.5%
	Model 2		21.0%
Blade thickness	Model 2	−10.98%	21.0%
	Model 3		21.7%
Position of splitter blades	Model 3	−5.63%	21.7%
	Model 4		22.0%

## Discussion

The blade angles of the baseline design were determined based on the conventional design theory of turbomachine and velocity triangles. However, one should note that the effective flowrates in the blade passages are higher than the inlet flowrates due to the secondary flow path. For the condition considered in this study (5 L/min, 360 mmhg), the average flowrates in the secondary flow path is around 50% of the inlet flowrate, i.e., around 2.5 L/min. It joined the main flow at the blade leading edge, led to significant disturbance of the main flow. It made the main flow highly three-dimensional and changed the velocity triangle. On the other hand, the flowrate of the secondary flow can hardly be reduced through redesigning the secondary flow path, which is constrained by factors such as the requirement of magnetic levitation and largely determined by the pressure head of the pump. Therefore, conventional design theory is no longer applicable to centrifugal blood pumps with the secondary flow path. This study focused on bringing down intensity of turbulence and secondary flows through design variations, to improve the hydraulic and hemolytic performance of centrifugal blood pumps. We show that turbulence and secondary flow intensities were very strong in the centrifugal blood pumps with secondary flow paths. Through changing design variables such as blade angles, blade thickness and position of splitter blades, turbulent intensities have been greatly reduced, the hydraulic and hemolytic performance of the pump model was considerably improved.

Another observation is that the geometric optimization had a greater impact on the hemolysis than the efficiency. Three of the hemolysis models employed in this study are power-law models where the power of effective stress is around or higher than two (1.9910, 1.9918, and 2.4160, respectively, cf. Table 4). On the other hand, efficiency is related to pressure loss, which is induced by both wall shear stress (proportional to pressure loss) and turbulent dissipation. Therefore, the change of stress level has more significant effects on efficiency than on hemolysis.

This study is among the first published studies on the design and optimization of centrifugal blood pumps with secondary flow path, with a focus on reducing turbulence intensities. Collectively, these results shed light on the impact of major design variables on turbulence intensity and pump performance, provide useful guidance to the design and optimization of centrifugal blood pumps.

This study also has limitations. Only certain design variables were considered. For each design variable, only two levels were investigated. Though the pump performance was indeed improved, the influence of an individual variable on pump performance (negative or positive) cannot be determined through this study. Optimization techniques such as RBF neural network and multi-objective genetic algorithm can achieve global optimal solutions, and have been widely used to optimize turbomachines. Nonetheless, these techniques normally need large group of samples. Since centrifugal blood pumps are generally not typical centrifugal pumps, empirical relationships between design variables and performance metrics generally do not exist. CFD simulation is basically the only way to build the samples. Thus, studies such as orthogonal experiment with more factors and levels are more practical for the optimization of centrifugal blood pumps with secondary flow path, and should be carried out in future research. Concerning blood compatibility, this study only considers hemolysis. This study shows that an extension of the splitter blade toward upstream decreased turbulence intensity and hemolysis level. However, the internal surface area of the pump was increased as well, which may increase the risk of platelet activation. A more complete metric of blood pump performance should be employed during the design optimization.

## Data Availability Statement

The raw data supporting the conclusions of this article will be made available by the authors, without undue reservation.

## Author Contributions

PW, JH, WD, CY, and SL: study concept and design. PW, JH, and WD: numerical simulation and data analysis. WD and SL: hydraulic experiment. JH, WD, and PW: drafting of the manuscript. CY, SL, and W-TW: critical revision of the manuscript. All authors contributed to the article and approved the submitted version.

## Conflict of Interest

The authors declare that the research was conducted in the absence of any commercial or financial relationships that could be construed as a potential conflict of interest.
